# ‘Manipulation’ without the parasite: altered feeding behaviour of mosquitoes is not dependent on infection with malaria parasites

**DOI:** 10.1098/rspb.2013.0711

**Published:** 2013-07-22

**Authors:** Lauren J. Cator, Justin George, Simon Blanford, Courtney C. Murdock, Thomas C. Baker, Andrew F. Read, Matthew B. Thomas

**Affiliations:** 1 Department of Entomology, Pennsylvania State University, University Park, PA 16802, USA; 2 Center for Infectious Disease Dynamics, Pennsylvania State University, University Park, PA 16802, USA; 3 Departments of Biology and Entomology, Pennsylvania State University, University Park, PA 16802, USA

**Keywords:** parasite manipulation, *Anopheles*, host-seeking, peripheral olfaction, foraging ecology, transmission

## Abstract

Previous studies have suggested that *Plasmodium* parasites can manipulate mosquito feeding behaviours such as probing, persistence and engorgement rate in order to enhance transmission success. Here, we broaden analysis of this ‘manipulation phenotype’ to consider proximate foraging behaviours, including responsiveness to host odours and host location. Using *Anopheles stephensi* and *Plasmodium yoelii* as a model system, we demonstrate that mosquitoes with early stage infections (i.e. non-infectious oocysts) exhibit reduced attraction to a human host, whereas those with late-stage infections (i.e. infectious sporozoites) exhibit increased attraction. These stage-specific changes in behaviour were paralleled by changes in the responsiveness of mosquito odourant receptors, providing a possible neurophysiological mechanism for the responses. However, we also found that both the behavioural and neurophysiological changes could be generated by immune challenge with heat-killed *Escherichia coli* and were thus not tied explicitly to the presence of malaria parasites. Our results support the hypothesis that the feeding behaviour of female mosquitoes is altered by *Plasmodium*, but question the extent to which this is owing to active manipulation by malaria parasites of host behaviour.

## 1. Introduction

Malaria's transmission is inextricably linked to the foraging and feeding behaviours of its insect vectors [1,2]. Since the 1980s, evidence has been collected suggesting that infection with *Plasmodium* parasites can alter mosquito behaviours (reviewed by Cator *et al.* [3]). Intriguingly, the nature of these changes appears dependent on the developmental stage of the parasite, with evidence for reduced foraging and feeding during the pre-infectious oocyst stage of infection [4–6] and increased foraging and feeding during the infectious sporozoite stage [4–6]. These observations have been interpreted as evidence for parasitic manipulation of mosquito behaviour [7,8], because reducing ‘risky’ feeding-associated activities during the non-infectious stages of parasite development (the highest daily rates of adult mosquito mortality are associated with finding and taking a blood meal [9]) and increasing probing and feeding at the infectious stages, is predicted to increase the overall likelihood of transmission [3]. If these behavioural alterations are due to parasite adaptations, whereby parasite genes encode traits which cause behavioural changes and have been favoured by natural selection because they do so, then this would be a classic case of an ‘extended phenotype’ [10–12].

The majority of evidence for behavioural alteration following infection with malaria focuses on ‘at-host’ foraging activities. At a range of less than 30 cm, Anderson *et al.* [5] demonstrated decreased biting persistence of female mosquitoes on a human host when infected with oocysts, and increased biting persistence by females when infected with sporozoites. Once on the host, studies have reported that sporozoite-infected females probe more frequently [4–6,13] and also take smaller blood meals [13], which could translate to multiple feeds per gonotrophic cycle [14,15]. To date, however, to our knowledge there have been no investigations of the effects of malaria infection on upstream feeding behaviours such as initiation of host-seeking, host orientation or host location. All these behaviours are strongly odour-mediated and research has shown that the peripheral olfactory system of malaria mosquitoes is highly malleable. For example, it is strongly responsive to ingestion of a blood meal [16,17] and can be altered by infection with fungal pathogens [18]. Whether *Plasmodium* infection also impacts olfaction and associated odour-related behaviours remains unknown.

To address these questions, we investigated the neurophysiological and behavioural responses to vertebrate host stimuli in the malaria mosquito *Anopheles stephensi* during different stages of infection with the malaria parasite, *Plasmodium yoelii*. Using this rodent model, we observed parasite stage-specific changes in the sensitivity of odorant receptors in the maxillary palps, which correlated with changes in long- and short-range attraction of female mosquitoes to hosts. These results are consistent with the manipulation hypothesis, while broadening current understanding of the phenotype. However, we also observed similar physiological and behavioural changes in mosquitoes that had taken an infectious blood meal but showed no positive signs of malaria infection, raising questions over the specificity of the response. In light of this, we examined the effects of a general immune challenge on behavioural and neurophysiological phenotypes. We found that activation of the mosquito immune system was sufficient to induce roughly equivalent changes in mosquito attraction and olfactory sensitivity to those associated with malaria infection. Our results strengthen the case for *Plasmodium* infection altering the behaviour of mosquitoes, but challenge the conventional notion of parasite manipulation.

## 2. Material and methods

### (a) Mosquitoes and *Plasmodium* infections

Eggs from over 1000 *An. stephensi* (NIH strain) females were placed in plastic trays (25 × 25 × 7 cm) filled with 1.5 l of distilled water. Upon reaching second instar, larvae were transferred to fresh trays at a density of 400 larvae per 1.5 l of distilled water. We fed larvae 10 mg of ground fish flakes (TetraFin, Melle, Germany) a day. We collected pupae and placed them in cages for emergence. Adults were provided with a 10 per cent glucose solution supplemented with 0.05 per cent para-aminobenzoic acid.

On day three post-emergence females were offered their first blood meal on an anaesthetized female mouse (C57 BL/6). One group of females received a blood meal from a mouse infected with 10^5^ *P. yoelii* parasites (clone 17XNL, from the World Health Organization Registry of Standard Malaria Parasites, University of Edinburgh, Edinburgh, UK) 4 days prior. Infected mosquitoes were tested on days 1–8 post-infection for oocyst-infected treatments and 9–28 days after infection for sporozoite-infected treatments (for exact days measured for each experiment, see below). Control females for all experiments were from the same rearing cycle that received an uninfected blood meal on the same day as females in the treatment group were offered an infected blood meal. At the conclusion of each behavioural assay, we dissected the midgut and salivary glands of each female from the infected treatment to determine infection status and to ensure that the proper stage of infection was measured. Females with oocysts in the midgut, sporozoites in the haemolymph or salivary glands were categorized as ‘infected’. Females that had taken an infectious blood meal, but did not have evidence of parasites were considered ‘exposed’. Infection prevalence and intensities for each experiment and replicate are listed in the electronic supplementary material, table S1.

### (b) Electropalpograms

Electrophysiological responses of the maxillary palps to 1-octen-3-ol were measured throughout the course of infection with *P. yoelii* following the methods of George *et al.* [18]. Briefly, electropalpogram (EPG) recordings were performed on mosquito maxillary palps using tungsten electrodes. A 10 μl aliquot from a 1 µg µl^−1^ hexane solution of 1-octen-3-ol was dispensed onto a standard 15 × 3 mm piece of Whatman filter paper, the hexane allowed to evaporate, and the paper then inserted into a 14-cm-long glass Pasteur pipette. Each odourant cartridge was thus loaded with 10 μg of odourant stimulus. Airborne puffs of the odourant were delivered to the palp preparation during the experiments via a 10 mm i.d. glass tube having a constant stream of charcoal-purified, humidified air passing through it and onto the preparation. Each odourant was puffed into the air stream through the Pasteur pipette odour cartridge, whose tip was inserted through a small hole in the airstream tube 11 cm away from its end. A stimulus flow-controller (Syntech, Hilversum, The Netherlands) delivered a 0.05 s pulse (2 ml) of air containing the volatiles into the air stream and onto the preparation. The EPG slow-potential (DC) responses to the stimuli were recorded and analysed using Syntech Autospike software (Syntech).

### (c) Experimental conditions for behavioural assays

All behavioural experiments were conducted 30 min after the insectary switched to the dark portion of the daily light cycle. Insectary conditions were set to 24°C with 80 per cent relative humidity. All females were deprived of sugar for 10–14 h prior to experiments. For the long-range host-seeking assay, experiments were conducted under low light (approx. 1 lux), whereas the short-range host-seeking experiments were conducted under red light.

### (d) Long-range host-seeking

Groups of 30 females which had received either a control or infected blood meal were transferred to a 30 × 30 × 30 cm mesh cage fitted with a remote release door. Each trial started when the release door was lifted and female mosquitoes were allowed to move into a 0.7 × 1.5 × 5 m mesh enclosure (see the electronic supplementary material, figure S1). A human host (L.J.C.) was positioned at the far end of the enclosure. Females were collected using a mouth aspirator as they approached the host. Each trial was conducted over a 1 h period. We aspirated females into individual cartons and noted time of capture. We also recorded the proportion of females exiting the release cage and the proportion approaching the host. The order of treatments (control blood meal or infected blood meal) was alternated on each evening to control for release order.

In the first replicate of the study, host-seeking assays were conducted one day prior to infection (pre-infectious) and then on day 6 for oocyst-stage infection and day 17 for sporozoite-stage infection. In the second and third replicates, females were tested on days 2, 4, 6, 8, 12, 15, 17 and 28 after a blood meal. After testing, the midgut and salivary glands of each female in the infected treatment were dissected and infection status and stage were assessed. We dissected midguts to detect oocysts-stage parasites on days 6–8 post-infection. Salivary glands were dissected the morning following testing. On the same day of dissections, the right wing of each female was removed and stored for measurement.

### (e) Short-range host-seeking

To investigate the effect of infection on short-range response to host cues, individual females were released into a 16 × 16 × 16 cm mesh cage. This release cage was fitted to a 48 cm clear plastic tube that was 12 cm in diameter (see the electronic supplementary material, figure S1). The other end of this tube opened into a ‘host cage’ (identical to the release cage), in which the hand of a human host (L.J.C.) was placed. A flap prevented test females from entering the tunnel prior to the start of the trial. Trials began when the flap was lifted and we recorded whether females responded to host cues and if so, the time it took to enter the host cage. Females failing to initiate active searching within 4 min were categorized as non-responders. We tested 25 females from each treatment group on days 6/7 (oocyst-stage) and days 14/15 (sporozoite-stage) for a total of 200 females per replicate. We rotated between treatment groups every five individuals to control for time of day effects. All treatment females were dissected to determine infection status. This experiment was replicated twice.

### (f) Immune-challenges

In order to determine whether the ‘manipulation’ phenotype was due specifically to exposure to *Plasmodium* parasites or to general immune challenge, we compared behavioural responses of females receiving a *Plasmodium-*infected blood meal with those challenged with heat-killed *Escherichia coli* directly following an uninfectious blood meal*.* Heat-killed *E. coli* has been shown to stimulate several immune pathways in the mosquitoes [19].

We divided 3–5-day-old females into two groups. One group received an infectious feed as described above. The other group received a blood meal from uninfected control mice. The females from this latter group were then further divided into four groups. The first group was anaesthetized briefly on ice and microinjected with 200 000 heat-killed *E. coli* (tetracycline resistance green fluorescent protein expressing dh5 alpha strain). The second group was anaesthetized on ice and injected with 0.2 μl of sterile Luria–Bertani's rich nutrient medium (LB, positive control for injury associated with injection). The third group was merely anaesthetized on ice (positive control for ice manipulation). The fourth and final group was an unmanipulated control.

To obtain heat-killed *E. coli*, we followed the methods in Murdock *et al.* [20]. Briefly, cultures in LB medium were grown overnight in a 37°C shaking incubator. We determined the injection dose of *E. coli* by reading the absorbance (OD_600_) from each dilution with a NanoDrop (Thermo Scientific, Wilimington, DE) and comparing it to a standard curve. *Escherichia coli* were heat-killed by autoclaving at 110°C for 25 min [20]. We compared the short-range host-seeking responses of females from the unmanipulated control, cold anaesthetization control, injury, *E. coli* challenged and *P. yoelii* challenged groups. Twenty-five females from each of the five treatments were tested on days 6–8 post-blood meal and days 14–16 post challenge to coincide with *Plasmodium* infection stages. The experiment was replicated twice.

### (g) Statistical analysis

Mean daily EPG amplitudes were compared on each day post-infection using a *t-*test with a Bonferroni correction in SAS (v. 9.2, SAS Institute Inc., Cary, NC). We used a general linear model to assess the effect of infection status on EPG amplitude within the females which received an infected blood meal.

We used a generalized linear model (GLM) fitted with a binary logistic regression in SPSS (v. 20, IBM, Armonk, NY) to determine the effect of infection on the likelihood that females approached the host in both the long-range and short-range assay. The significance of treatment (infected blood meal/control blood meal), stage (days corresponding with oocyst/sporozoite infection), replicate, release order and wing length parameters were assessed. In order to determine the effect of exposure (receiving a blood meal from an infected mouse) versus infection (harbouring malaria parasites at the time of dissection), we ran the model on the females from the infected treatment alone and instead of treatment group tested the effect of infection status (infection/exposed).

We also used a GLM fitted with a binary logistic regression to determine the effect of immune challenges on the likelihood that females were attracted to the host. In this case, we determined the statistical significance of treatment (control/cold anaesthetized/sham injected/heat-killed *E. coli* injected/*Plasmodium* challenged), stage (periods corresponding with oocyst infection (days 6–8) and sporozoite infection (days 14–16)) and wing length on the likelihood of a positive response using a binary logistic regression. In order to compare responses between the two stages and within a treatment group, we ran the model for each treatment group separately. We additionally ran the model separately with only the groups challenged with heat-killed *E. coli* and *P. yoelii*, the heat-killed *E. coli* and injury control group, and the injury control group and heat-killed *E. coli* group to further characterize the relationships between these treatments.

In all cases, full models were reduced through stepwise elimination of non-significant interactions and terms. Significance values reported for significant terms were those taken from the final model. Significance values reported for non-significant terms were those computed in the final step prior to removal of the term from the model. Non-significant values were only reported if they had a significant interaction with another parameter. All data were deposited in the Dryad Repository: doi:10.5061/dryad.j4n89.

## 3. Results

### (a) The effect of infection on palp sensitivity

**Figure 1.**
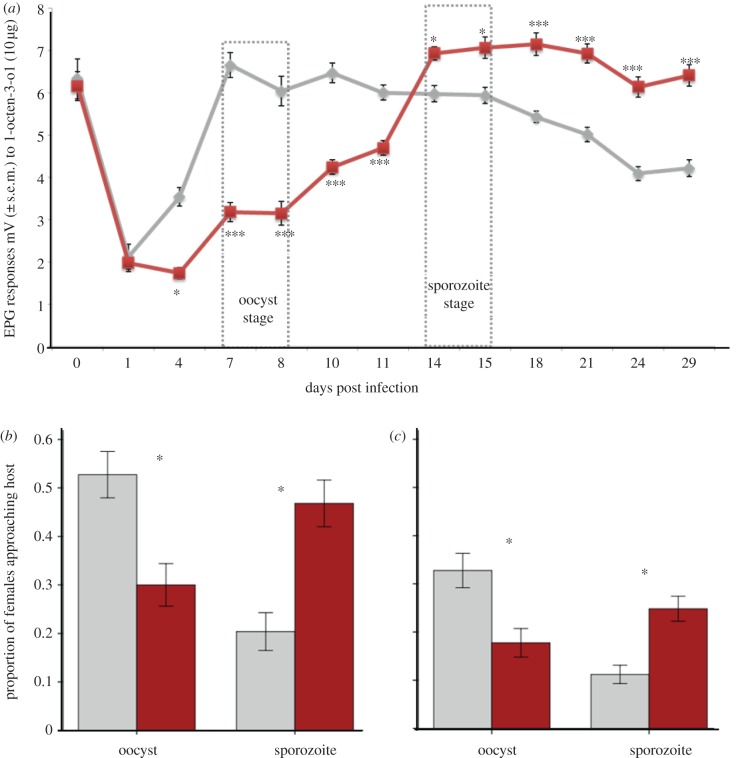
The effect of *Plasmodium yoelii* infection on neurophysiological and behavioural responses of female *Anopheles stephensi* to host stimuli. (*a*) Line graph showing EPG responses during different stages of *P. yoelii* development within *An. stephensi* mosquitoes. The light grey line denotes EPG responses to 1-octen-3-ol (10 μg) in blood-fed, uninfected *An. stephensi* females, and the dark grey/red line represents EPG responses of blood-fed, infected females. EPGs were suppressed during the non-transmissible oocyst stage of the parasite and enhanced during the transmissible sporozoite stage. *p < 0.05; **p < 0.01; ***p < 0.001. (*b*) The effect of an infected blood meal on the proportion of females approaching a human host in the long-range host-seeking assay. Females received either a control (light grey) or infected (dark grey/red) blood meal. The oocyst stage represents days 6–8 post-blood meal and the sporozoite stage represents 11–28 days post-blood meal. Error bars represent ± 1 s.e. and an asterisk indicates significance *p < 0.05. (*c*) Proportion of females attempting to feed on a human host in the short-range host-seeking assay. Oocyst stage measurements were taken on days 6–7 and sporozoite stage measurements were taken on days 14–15. Error bars represent ± 1 s.e. and an asterisk indicates significant difference as indicated by the Wald Chi-squared statistic in binary logistic regression (*p* < 0.05). (Online version in colour.)

Females from the infected and control groups all exhibited equally high EPG amplitudes in response to 1-octen-3-ol on day 0, before they were offered a blood meal (*n* = 8, *t* = 0.458, *p* = 0.624; figure 1*a*). Once the females had been blood-fed, EPG amplitudes decreased, and females from both infected and uninfected blood meal groups exhibited relatively low EPG amplitudes on day 1 post-feeding (*n* = 8, *t* = 0.363, *p* > 0.05). By day 4, EPG amplitudes from control females began to increase and were significantly higher than those from the infected group (*n* = 8, *t* = 4.72, *p* < 0.001; figure 1*a*). This pattern continued with infected females exhibiting significantly lower EPGs (*n* = 9, *t* = 6.92, *p* < 0.001) throughout the nominal oocyst stage up to days 10–11 (figure 1*a*). After day 14, EPG amplitudes in the infected group increased. This increase corresponded with the migration of the sporozoites to the salivary glands and the females becoming infectious. During this same period, control females began to show a gradual decrease in EPG amplitude possibly owing to senescence [21]. This resulted in the EPG amplitudes of infected females becoming significantly higher than control females (*n* = 10, *t* = 5.69, *p* < 0.001; figure 1*a*). Similar results were found for different concentrations of 1-octen-3-ol and other known host odours (see the electronic supplementary material, figures S2–S4).

### (b) The effect of malaria-infected blood meal on host-seeking

The differences in electrophysiological response were paralleled by behavioural responses in the both long- and short-range assays. In the long-range assay, we confirmed that females assessed prior to the blood meal were equally as likely to respond to the host (*n* = 78, Wald *χ*^2^ = 1.30, *p* = 0.25). Females fed on infected blood were less likely to approach the host at the oocyst stage and more likely to approach the host at the sporozoite stage than their age-matched controls (figure 1*b*,*c*; treatment by malaria-stage interaction: long-range assay: *n* = 898, Wald *χ*^2^ = 16.859, *p* < 0.001; short-range: *n* = 438, Wald *χ*^2^ = 31.40, *p* < 0.001).

### (c) The effect of infection status on olfactory sensitivity and host-seeking

The malaria dissection data (visual assessment of oocyst and sporozoite prevalence and intensity) did not indicate that infection status affected the likelihood that mosquitoes fed on infected blood exhibited the altered phenotype. Females that fed on malaria-infected blood but in which parasites were not detected were as likely to display the altered phenotype as those with detectable parasites (electropalpgram recordings: *n* = 38, Wald *χ*^2^ = 0.47, *p* = 0.49, the likelihood of recruiting to the human host in the short-range assay: *n* = 200, Wald *χ*^2^ = 8.61, *p* = 0.13; long-range assay: *n* = 486, Wald *χ*^2^ = 1.04, *p* = 0.90).

### (d) The effect of immune challenge on olfactory sensitivity and host-seeking

**Figure 2.**
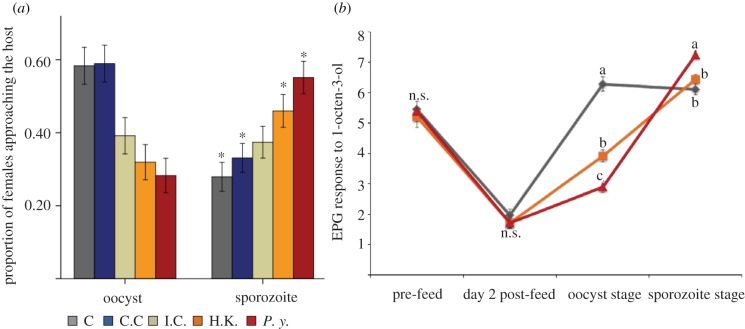
The effect of immune challenge on host-seeking and electropalpogram (EPG) response. (*a*) Comparing the proportion of females approaching the host during test periods associated with oocyst and sporozoite stage infection. ‘C’ is unmanipulated control group; ‘C.C.’ is cold anaesthetization control; I.C. is injury control, ‘H.K.’ is the group challenged with heat-killed *E. coli*; and ‘*P.y.*’ the group challenged with a *P. yoelii-*infected blood meal. Asterisk indicates significant increase or decrease in comparison with the same treatment during the first test period. (*b*) EPG responses (mV ± s.e.) corresponding to different stages of *P. yoelii* development within *An. stephensi* females. The ‘oocyst stage’ measurements were taken on days 7 and 8 post-blood meal and the ‘sporozoite’ stage measurements were taken on days 14 and 15 post-blood meal. The grey line (diamond markers) denotes EPG responses to 1-octen-3-ol (10 μg) in blood-fed, uninfected *An. stephensi* females and the light grey/orange line (square markers) represents the EPG responses of heat-killed *E-coli* infected female mosquitoes. The dark grey/red line (triangle markers) indicates the EPG responses of malaria-infected female mosquitoes. ANOVAs were performed using SAS v. 9.2. Individual comparisons were made between treatments using *t*-tests within each day, and Bonferroni correction was used. Means on each day having no letters in common are significantly different according to a *t*-test (*p* ≤ 0.05). (Online version in colour.)

Mosquitoes challenged with heat-killed *E. coli* directly after taking an uninfectious blood meal and *P. yoelii-*infected mosquitoes exhibited similar phenotypes (figure 2*a*).

When we compared the response in the two test periods within a treatment, females in both unmanipulated and cold anaesthetized control groups were less likely to respond in the later test period (Wald *χ*^2^ = 314.17, *p* = 0.001, Wald *χ*^2^ = 18.80, *p* < 0.001, respectively). The response of the injury control did not vary significantly between the two test periods (Wald *χ*^2^ = 0.07, *p* = 0.78). Females challenged with heat-killed *E. coli* showed the ‘manipulation phenotype’ and, similar to the females challenged with *P. yoelii*, these females exhibited a significantly greater attraction response during the later test period (heat-killed *E. coli*, *n* = 265, Wald *χ*^2^ = 4.36, *p* = 0.037, *P. yoelii*, *n* = 269, Wald *χ*^2^ = 15.13, *p* < 0.001). When we further investigated the relationships, we found that the response across stages for the *P. yoelii* challenged group was similar to the group challenged with heat-killed *E. coli* (test period × group interaction, Wald *χ*^2^_2_ = 1.75, *p* = 0.185), but significantly different from the injury control group (test period × group interaction, Wald *χ*^2^_1_ = 8.99, *p* = 0.003). The heat-killed *E. coli* group response was not significantly different from that of the injury control (time period × group interaction, Wald *χ*^2^_1_ = 3,37, *p* = 0.07).

The EPG responses of the heat-killed *E. coli* group also mirrored the malaria-exposed group, both showing significantly lower EPG amplitudes than controls at 6–7 days post-blood meal (*n* = 14, *t* = 12.60, *p* < 0.001; figure 2*b*) and rebounding beyond the controls by days 14 and 15 (figure 2*b*). However, while qualitatively similar, the magnitude of the rebound in the *E. coli* group was slightly less than the malaria group and did not differ significantly from the controls (*n* = 16, *t* = 1.33, *p* > 0.05; figure 2*b*).

## 4. Discussion

Our observations expand the behavioural changes associated with malaria parasite infection to include stage-specific changes in host-seeking and attraction. Female mosquitoes carrying the pre-infectious oocyst stage of the parasite were less likely to be attracted to a host, whereas those at the infectious sporozoite stage became more likely to approach the host and attempt to feed. These stage-specific behavioural changes were paralleled by changes in the sensitivity of the mosquito olfactory neurons. Lefevre *et al.* [22] determined that the head proteome of *Anopheles* females infected with *Plasmodium berghei* contained proteins not found in uninfected females. Some of these proteins were associated with regulation of the insect central nervous system (CNS; [22]). Other studies also report effects of various parasites on interneuron activity and electrical excitability of neurons in the CNS [23]. However, ours is the first example, as far as we are aware, of stage-specific alterations in the sensitivity of peripheral sensory neurons in any parasite–host manipulation system.

Unexpectedly, we observed the behavioural phenotype and components of the neurophysiological phenotype in both females definitively containing malaria parasites and those simply exposed to an infectious blood meal (females that either did not pick up parasites or cleared the infection at a very early stage). Given that we determined infection status using microscopy, it is possible that some females with low-intensity infections could have been incorrectly classified as uninfected. However, we also found that general immune stimulation with heat-killed *E. coli* generated equivalent ‘stage-specific’ changes in host-seeking and produced similar changes in the sensitivity of the maxillary palp olfactory neurons. There were some small quantitative differences between the EPGs of the malaria-infected and immune-challenged females, which could indicate that the parasite is impacting the mosquito in a different way to *E. coli*. Alternatively, these differences could be owing to variation in the strength or nature of immune response triggered by the respective challenges (it would be surprising if our single heat-killed *E. coli* challenge was exactly equivalent to the *P. yoelii* challenge). Such quantitative differences notwithstanding, there was a striking qualitative similarity between the EPGs of females challenged with heat-killed *E. coli* and *Plasmodium.* This observation suggests that while altered mosquito behaviour may be a product of parasite interaction with the immune system, the pathways used are not uniquely stimulated by the parasite.

A number of behaviours have been assigned to the ‘manipulation phenotype’. ‘At-host’ behaviours such as probing and engorgement success have been found to be altered with malaria infection [4,6,13]. These behaviours have been mechanistically explained by decreased production of apyrase, an enzyme important for feeding efficiency [4]. There is also evidence that infection modifies the threshold at which females reach satiation, causing infected females, which are already taking longer to engorge owing to difficulty probing, to take smaller blood meals [13]. Decreased blood meal size could lead to multiple blood meals from multiple hosts per gonotrophic cycle [14]. Additionally, others have reported that the head proteome [22] and salivary protein profiles [24] differ between infected and uninfected females. The involvement of the peripheral nervous system and immune response reported in this study offer new insights into potential mechanisms underlying these altered phenotypes.

In our experiments, injury alone was not sufficient to trigger an altered phenotype equivalent to *Plasmodium* challenge, whereas immune activation by heat-killed *E. coli* and exposure to malaria parasites (independent of whether parasites actually established) produced roughly equivalent changes in behaviour and neurophysiology. There was a partial response from the injury control group that was statistically similar to the heat-killed *E. coli* challenge (this might be expected as the heat-killed *E. coli* challenge also included injury), but this did not match the *P. yoelii* treatment indicating an additional role of parasite/pathogen challenge. Thus, at least some of the changes we observe appear linked to immune stimulation. This observation does not negate the possibility that the behavioural changes observed with infection are parasitic manipulation. It can be extremely difficult to separate host from parasite adaptations as causes of altered behavioural phenotypes, and many parasites rely on host-mediated changes in behaviour to enhance transmission [23]. However, given that the presence of *Plasmodium* parasites was not required for manifestation of the altered behavioural phenotype, it seems unlikely that they are directly manipulating the mosquito via constant secretion of neuromodulators or other molecules that require their physical presence in key tissues/organs like the brain [23], or that if they are, they are manipulating pathways readily stimulated by antigens alone. There is extensive interaction between the nervous and immune system [25] and these connections are often exploited in parasitic manipulation [23]. Furthermore, given the apparent general nature of the response and that other insects adaptively respond to immune challenge by altering feeding behaviours [26], our findings also do not exclude a potential role of an adaptive host response as a driver of this phenotype.

Whether or not the suite of behavioural changes associated with malaria infection is parasite manipulation, host response or some combination thereof, our results support the hypothesis that malaria parasite-infected females exhibit altered feeding phenotypes. Given the altered phenotypes appeared linked to a highly generalized response to infection, we expect our results to extend to other mosquito species challenged with human malaria. Even modest effects on these aspects of mosquito behavioural ecology could have important consequences for transmission. All else being equal, a 20 per cent reduction in feeding-associated mortality during the pre-infectious stage (this is the magnitude of response we observed in the long-range host-seeking assay) could increase the relative force of malaria infection by 60 per cent [3]. Such impacts highlight the need to better characterize the foraging and feeding behaviour of malaria mosquitoes and in particular, the behaviour of the small proportion of the mosquito population actually responsible for transmission.
